# The effects of early aggressive therapy in JIA: results of the TREAT study

**DOI:** 10.1186/1546-0096-10-S1-A56

**Published:** 2012-07-13

**Authors:** Carol A Wallace, Edward H Giannini, Steven J Spalding, Philip J Hashkes, Kathleen M O’Neil, Andrew S Zeft, Ilona S Szer, Sarah M Ringold, Hermine Brunner, Laura E Schanberg, Robert P Sundel, Diana Milojevic, Marilynn G Punaro, Peter Chira, Beth S Gottlieb, Gloria C Higgins, Norman T Ilowite, Yukiko Kimura, Bin Huang, Daniel J Lovell

**Affiliations:** 1 Children's Hospital and Regional Medical, Seattle, WA, USA; 2 Children'sHospital Montefiore, Bronx, NY, USA; 3 Childrens Hospital Medical Center, Boston, MA, USA; 4 Cincinnati Children's Hospital Medical Center, Cincinnati, OH, USA; 5 Cleveland Clinic, Cleveland, OH, USA; 6 Duke University Medical Center, Durham, NC, USA; 7 Hackensack University Medical Center, Hackensack, NJ, USA; 8 Nationwide Childrens Hospital, Columbus, OH, USA; 9 Oklahoma University Health Science Center, Oklahoma City, OK, USA; 10 Rady Childrens Hospital San Diego, San Diego, CA, USA; 11 Schneider Children's Hospital, New Hyde Park, NY, USA; 12 Shaare Zedek Medical Center, Jerusalem, Israel, USA; 13 Stanford University School of Medicine, Stanford, CA, USA; 14 Texas Scottish Rite Hospital, Dallas, TX, USA; 15 UCSF, San Francisco, CA, USA; 16 University of Utah, Salt Lake City, UT, USA

## Purpose

Early aggressive therapy has been shown to result in superior outcomes in adults with RA, but evidence for a similar benefit has not been demonstrated in children with JIA. The objective was to determine if aggressive treatment initiated within the first 12 mos after onset of extended oligoarticular (e-oligo), or RF + or (-) polyarticular JIA (poly JIA) can induce inactive disease (ID) within 6 mos (primary endpoint) and clinical remission on medication (CRM: ID for 6 continuous mos on medication; exploratory endpoint) within 1 yr of starting therapy.

## Methods

TREAT was designed as a multi-centered, prospective, double blind, randomized, placebo controlled clinical trial in children aged 2 to 16 yrs with e-oligo or poly JIA of ≤ 12 mos duration. Participants were randomized 1:1 into 1 of 2 aggressive treatment arms: (Arm 1) MTX 0.5 mg/kg/wk SQ (40 mg max), plus etanercept 0.8 mg/kg/wk (50 mg max), plus prednisone 0.5 mg/kg/d (60 mg max) tapered to 0 by 17 wks or; (Arm 2) MTX (same dose as Arm 1) plus etanercept placebo, plus prednisolone placebo, then followed on protocol for up to 12 mos. After 4 mos on therapy participants who failed to achieve at least an ACR Pediatric 70 received open label etanercept, MTX, and prednisolone in the same doses as Arm 1. At 6 mos the participants who achieved ACR Pediatric 70 but failed to achieve ID received open label medication as in Arm 1. Efficacy analyses focused on the intent-to-treat approach. Safety data were recorded for all participants who received at least one dose of medication.

## Results

15 centers enrolled 85 participants (64 [75%] female) with a median age of 11.1 yrs and disease duration of 4.1 mos. 70% were ANA+, 33% RF+, and 33% anti-CCP+. At baseline the median physician’s global assessment of disease activity and parent global assessment of overall well-being were 7 and 5 respectively. There were no significant inter-Arm differences in any of these variables. At baseline, Arm 2 had a statistically higher mean ESR (45 vs. 29 mm/hr) and active joint count (25.5 vs. 18.9) compared to Arm 1. By 4 mos, 30 of 42 (71%) participants in Arm 1 and 19 of 43 (44%) in Arm 2 achieved an ACR Pediatric 70 (X2 = 6.5; p<.011). By 6 mos, 17 of 42 (40%) of participants in Arm 1 achieved ID, compared to 10 of 43 (23%) in Arm 2 (X2 = 2.91; p = .088). Although all 6 ACR pediatric core set variables showed highly significant improvement from baseline by 6 mos in both Arms (p < .001), 5 of 6 core set variables showed statistically greater improvement in Arm 1 vs. Arm 2. By 12 mos, 12 (14%) participants achieved CRM; 9 (21%) had remained in Arm 1, and 3 (6%) had remained in Arm 2 throughout the study (p = 0.0534). There were no significant inter-arm differences in the incidence of Grade 3 or higher adverse events, including infections requiring systemic therapy. There were 3 SAEs: pneumonia (Arm 1), psychosis (open label), and bacteremia with septic arthritis (open label). All resolved without sequelae.

## Conclusion

Although this trial did not reach its primary endpoint, early aggressive therapy in this cohort of children with severe JIA and a high rate of RF positivity resulted in substantial proportions of participants achieving an ACR Pediatric 70 by 4 mos, ID by 6 mos, and CRM within 12 mos of treatment initiation.

## Disclosure

Carol A. Wallace: Amgen Inc., 2; Edward H. Giannini: None; Steven J. Spalding: None; Philip J. Hashkes: None; Kathleen M. O’Neil: None; Andrew S. Zeft: None; Ilona S. Szer: None; Sarah M. Ringold: None; Hermine Brunner: None; Laura E. Schanberg: None; Robert P. Sundel: None; Diana Milojevic: None; Marilynn G. Punaro: None; Peter Chira: None; Beth S. Gottlieb: Pfizer Inc, 5; Gloria C. Higgins: None; Norman T. Ilowite: None; Yukiko Kimura: None; Bin Huang: Amgen Inc., 2; Daniel J. Lovell: Abbott Laboratories, 9, Amgen Inc., 5, Bristol-Myers Squibb, 9, Centocor, Inc., 9, Hoffmann-La Roche, Inc., 9, Novartis Pharmaceuticals Corporation, 9, Regeneron Pharmaceuticals, Inc., 9, UBC, 9.

